# Energy cost of ambulation in trans-tibial amputees using a dynamic-response foot with hydraulic versus rigid ‘ankle’: insights from body centre of mass dynamics

**DOI:** 10.1186/s12984-019-0508-x

**Published:** 2019-03-14

**Authors:** Graham N. Askew, Laura A. McFarlane, Alberto E. Minetti, John G. Buckley

**Affiliations:** 1 School of Biomedical Sciences, Faculty of Biological Sciences, University of Leeds, Leeds, LS2 9JT UK; 2 Division of Biomedical Engineering, School of Engineering, University of Bradford, Bradford, BD7 1DP UK; 3 Department of Pathophysiology and Transplantation, Faculty of Medicine, University of Milan, Milan, Italy

## Abstract

**Background:**

Previous research has shown that use of a dynamic-response prosthetic foot (DRF) that incorporates a small passive hydraulic ankle device (*hy*A-F), provides certain biomechanical benefits over using a DRF that has no ankle mechanism (*rig*A-F). This study investigated whether use of a *hy*A-F in unilateral trans-tibial amputees (UTA) additionally provides metabolic energy expenditure savings and increases the symmetry in walking kinematics, compared to *rig*A-F.

**Methods:**

Nine active UTA completed treadmill walking trials at zero gradient (at 0.8, 1.0, 1.2, 1.4, and 1.6 of customary walking speed) and for customary walking speed only, at two angles of decline (5° and 10°). The metabolic cost of locomotion was determined using respirometry. To gain insights into the source of any metabolic savings, 3D motion capture was used to determine segment kinematics, allowing body centre of mass dynamics (BCoM), differences in inter-limb symmetry and potential for energy recovery through pendulum-like motion to be quantified for each foot type.

**Results:**

During both level and decline walking, use of a *hy*A-F compared to *rig*A-F significantly reduced the total mechanical work and increased the interchange between the mechanical energies of the BCoM (recovery index), leading to a significant reduction in the metabolic energy cost of locomotion, and hence an associated increase in locomotor efficiency (*p* < 0.001). It also increased inter-limb symmetry (medio-lateral and progression axes, particularly when walking on a 10° decline), highlighting the improvements in gait were related to a lessening of the kinematic compensations evident when using the *rig*A-F.

**Conclusions:**

Findings suggest that use of a DRF that incorporates a small passive hydraulic ankle device will deliver improvements in metabolic energy expenditure and kinematics and thus should provide clinically meaningful benefits to UTAs’ everyday locomotion, particularly for those who are able to walk at a range of speeds and over different terrains.

## Background

Individuals who have undergone amputation of one (or both) of their lower limbs utilize movement adaptations and/or compensatory joint kinetics in order to walk using a prosthetic device over which they have only indirect control. The compensatory actions utilized by unilateral trans-tibial amputees (UTA) include an increase in mechanical power generation, at each hip during both early and late stance, and at the ankle of the intact limb in late stance [1–4]. As a result of such biomechanical adaptations, as well as the fact that no net mechanical power is generated by passive (i.e. non-powered) prosthetic foot-ankle devices, the metabolic cost of ambulation is higher in UTA than in able-bodied individuals when walking at comparable speeds [5–8]. As a consequence, UTA tend to choose a customary walking speed that is slower than that chosen by able-bodied individuals [6, 9, 10]. Thus the metabolic energy expenditure to travel a given distance is significantly higher in UTA than in age-matched able-body controls [6, 11, 12]. In addition, asymmetries in the dynamics of locomotion may also require a higher metabolic cost of locomotion [13]. Accordingly, reducing the metabolic cost of locomotion and maximising gait symmetry have been, and remain, key criteria for developments in prosthetic design. Arguably the most significant prosthetic development has been the introduction of dynamic response feet (DRF): sometimes referred to as ‘energy storing and return’ feet. Typically, such feet incorporate carbon-fibre heel and forefoot keels that elastically deform during loading and subsequently recoil (return energy) to aid forwards progression. Use of such feet has been shown to increase the amount of late stance mechanical power return at the distal end of the prosthetic shank (‘ankle’) [2, 5, 14] and as a result the metabolic cost of locomotion is reduced compared to when using traditional semi-rigid type feet [15, 16]; though such reductions have not always been found [5, 8, 11].

Most (> 85%) types of DRF currently available are secured to the prosthetic shank via a non-articulating means of attachment (i.e. they have no ‘ankle’). However, when walking using such feet the forces applied at the heel following contact with the ground cause the heel keel to deform, creating simulated plantar-flexion; lowering the toe region of the foot towards the floor. As the centre of mass moves forward over the foot, body weight is shifted to the forefoot keel, which deforms to produce simulated dorsiflexion. When UTA descend slopes, deflexion of the heel keel may not be great enough to ensure the toe-region of the foot makes contact with the inclined floor surface, and hence compensatory actions are required, not only to gain a ‘foot flat’ position but also to modulate how quickly the shank (and thus the body’s centre of mass, BCoM) rotates forward over the foot [17–19]. Consequently, walking down slopes is problematic for amputees. Accordingly, ‘ankle’ devices that allow a limited range of stance-phase (passive) articulation between the foot and shank have been added to some DRF. Recently a DRF incorporating a small hydraulic ‘ankle’ device that provides 9° of passive articulation has become clinically available (*Echelon*; Endolite, Chas. A Blatchford & Sons). We have shown that when UTA walk over level ground using such a device compared to using an identical DRF with a rigid ‘ankle’, body weight is transferred onto the prosthetic limb in a smoother less faltering manner [20], with accompanying smaller reduction in body centre of mass forward speed [21], and reduced compensatory intact-limb joint work per meter travelled [1]. These findings suggest there might be a metabolic cost saving when walking using such a device, although given that the ankle device adds around 400 g to a foot’s mass, this might not necessarily be the case. Moreover, because the device facilitates the foot/ankle going through a greater range of motion, its use may be particularly beneficial for walking over a range of speeds and down slopes. Therefore, the purpose of the present study was to determine how the metabolic costs of treadmill walking at different speeds and at different angled declines, was effected in UTAs when they switched from using an DRF with non-articulating rigid attachment (*rig*A-F, Esprit; Chas. A Blatchford & Sons), to using the same DRF but incorporating a hydraulic ‘ankle’ attachment (*hy*A-F; Echelon). In an attempt to provide insights into the source of any metabolic savings, the study also determined how inter-limb asymmetry, internal and external mechanical work, and locomotor efficiency were affected by using the *hy*A-F compared to *rig*A-F.

## Methods

### Participants

Nine male, physically active UTA (mean ± SD; age 41.3 ± 14.3 years, mass 79.6 ± 13.3 kg, height 178.6 ± 6.6 cm) took part, each giving written informed consent prior to their involvement. All had undergone amputation at least 2 years prior to participation (mean 12.9 ± 13.7 years, range 2 to 45 years) and all had used their current prosthesis for at least 6 months. All participants habitually used an Esprit foot (Chas. A. Blatchford and Sons Ltd., Basingstoke, UK), which has a rigid ‘ankle’ (*rig*A-F). The study was conducted in accordance with the tenets of the Declaration of Helsinki and local bioethics committee approval was obtained.

### Protocol and prosthetic intervention

Participants completed treadmill (pulsar 3p, h/p/cosmos, Nussdorf-Traunstein, Germany) walking trials at five different speeds at zero gradient (equivalent to 0.8, 1.0, 1.2, 1.4, and 1.6 of customary walking speed) and for customary walking speed only, at two angles of decline (5° and 10°). Each participant’s customary walking speed was determined using a ‘staircase’ procedure [22], and was determined using the *rig*A-F. Trials were undertaken in two blocks; one block was undertaken using a *rig*A-F and the other using a *hy*A-F. Attachment type order was randomised and, for each attachment condition, the five speed levels (at zero gradient) and the two decline gradients (at customary speed) were completed in random order.

We are aware that treadmill walking is known to cause subtle changes in gait [23, 24] and metabolic energy expenditure compared to walking overground [25]. However, we think it is unlikely that differences measured between the foot type conditions during treadmill walking would be fundamentally different from walking overground; hence we chose a treadmill approach for practical reasons.

Prior to completing the block using the *hy*A-F each participant’s prosthesis was altered by exchanging the existing *rig*A-F device for a *hy*A-F. All alterations were made by the same experienced prosthetist. Everything about the alignment and set-up of the prosthesis was kept as near to constant as possible when one attachment type was exchanged for the other: the socket, suspension, overall length of the prosthesis and alignment of the shank pylon were unchanged across attachment types. Because the *rig*A-F and *hy*A-F each have an identical foot section and each use the same means of attaching to the prosthetic shank-pylon, when switching from one foot to the other, the same foot alignment (set-up) was easily maintained because the only change required was an alteration in shank length which was made by either including a shorter (*hy*A-F) or longer (*rig*A-F) pylon section.

The *hy*A-F hydraulic dashpot’s compression (plantar-flexion) and extension (dorsi-flexion) rates (hydraulic resistance) were heuristically fine-tuned using observations of the participant’s gait and their feedback whilst they walked overground at their self-selected customary walking speed. Once the *hy*A-F was fitted and adjusted, participants walked around the laboratory and on the treadmill at a range of speeds to become accommodated. For participants using the *rig*A-F first (block 1), trials were completed without any adjustment to their prosthesis. For participants using the *rig*A-F second (block 2), the original set-up of their prosthesis was reinstated following completion of block 1 (undertaken with *hy*A-F), and participants were given an accommodation period (30–40 min) in order to reacquaint themselves with their habitual prosthesis.

### Metabolic cost of locomotion

Participants, wearing their own flat-soled shoes and shorts, were fitted with a silicon face mask and breathed via a two-way, non-rebreathing, low resistance valve. Exhaled gases passed through a respiratory flow head and a subsample passed through a carbon dioxide and oxygen analyser (Foxbox, Sable Systems International, Las Vegas, NV, USA). Resting oxygen uptake and carbon dioxide production was measured while the participant stood quietly wearing the prosthesis being tested. During experimental measurements, participants walked at each speed until the rate of oxygen consumption $\left({\dot{V}}_{O_2}\right)$ and carbon dioxide production $\left({\dot{V}}_{CO_2}\right)$ had plateaued. ${\dot{V}}_{O_2}$ and ${\dot{V}}_{CO_2}$ were determined in the final 1–2 min of each trial (which lasted approximately 7 mins). The respiratory exchange ratio (*RER*) was calculated as the ratio of ${\dot{V}}_{CO_2}$ to ${\dot{V}}_{O_2}$ and was less than 1 in all cases, indicating aerobic metabolism. *RER* was used to convert the metabolic cost of locomotion from ml O_2_ into J [26]. The net mass-specific metabolic cost of locomotion (*C*_met_; J kg^− 1^ m^− 1^) was calculated as the ratio between the difference between the exercising, steady-state and standing metabolic rates and mean speed.

In between each ambulatory condition participants took a standing rest until their heart rate had dropped to within 10% of their baseline level. Participants rested for approximately 1 h before completing the second data collection block.

### Kinematics

Body segment kinematics were determined by tracking body segment coordinates at 100 Hz using a twelve camera, 3D motion capture system (Oqus 4, Qualysis AB, Göteborg, Sweden). Eighteen reflective markers were positioned bilaterally on the body (or equivalent locations on the prosthesis) in the following locations: anterior to ear tragus, shoulder, elbow, wrist, greater trochanter, lateral condyle of the femur, lateral malleolus, calcaneus, and 5th metatarsal head. This allowed determination of twelve body segments [27], including the 3D position of the centre of mass of each segment (determined from anthropomorphic tables for body segments [28, 29] and measured in the prosthesis). From the segment CoM locations, the 3D trajectory of the BCoM was computed [30]. Kinematics were recorded during the final two minutes of the trial, once oxygen consumption and carbon dioxide production had plateaued.

The trajectory of BCoM was represented as a 3D closed loop contour, or Lissajous plot, representing its displacement relative to the average position [30–32]. A Fourier series with ten harmonics was fit to the positional data in the forward, vertical and lateral directions with time as the independent variable. The average 3D Lissajous contours were determined in the form:

$$ \overline{\widehat{x}}(t)=\sum \limits_{i=1}^{10}{\overline{c}}_i^x\sin \left( it+{\overline{\phi}}_i^x\right) $$

$$ \overline{\widehat{y}}(t)={\overline{a}}_0^y+\sum \limits_{i=1}^{10}{\overline{c}}_i^y\sin \left( it+{\overline{\phi}}_i^y\right) $$

$$ \overline{\widehat{z}}(t)=\sum \limits_{i=1}^{10}{\overline{c}}_i^z\sin \left( it+{\overline{\phi}}_i^z\right) $$

where *x*, *y* and *z* are the forward, vertical and lateral directions, respectively, *t* is time (normalised to 2π radians), *i* is the harmonic number, ${\overline{c}}_i$ and *ϕ* are the harmonic coefficient (amplitude) and phase angle, respectively; and $\overline{}$ denotes the average of the predicted ($\widehat{}$) value. The symmetry between the intact- and prosthetic- limb steps was calculated as the Dynamic Symmetry Indices along each axis (*SI*^*x*^, *SI*^*y*^, *SI*^*z*^), determined from the coefficients of the 10-harmonic Fourier series (following [32]; *SI*, 0: no symmetry between prosthetic and intact steps, 1: complete symmetry).

Note that in order to allow average Lissajous plots to be computed, the Fourier series for left leg amputees were reflected to make all participants effectively right-legged amputees.

### Mechanical work and locomotor efficiency

The overall internal mechanical work was calculated as the sum of the internal work of each limb and the head-trunk. The instantaneous translational kinetic energy of each segment was determined from the segment’s speed relative to the BCoM speed (KE_t_). The instantaneous rotational kinetic energy was determined from the segment’s absolute angular velocity (KE_r_). The sum of the translational and rotational kinetic energies for each segment within the same limb and within the head-trunk were summed to yield the kinetic energy (KE) of each limb and of the head-trunk. The positive increments in KE of each limb and head-trunk were summed to obtain the internal mechanical work of each limb and of the head-trunk. The overall internal mechanical work (*W*_INT_) was calculated as the sum of the internal work of each limb and the head-trunk (following [33]). The total mechanical energy of the BCoM (*E*_CM_) was calculated as the sum of the instantaneous kinetic and potential energy of the BCoM. The external mechanical work (*W*_EXT_) was calculated by summing the positive increments in *E*_CM_. The total mechanical work (*W*_TOT_) is the sum of *W*_INT_ and *W*_EXT_. All measurements of mechanical work were expressed as a mass-specific cost of locomotion (J kg^− 1^ m^− 1^).

The potential degree of interchange between the potential and kinetic energy of the centre of mass was quantified by calculating the recovery index (*R*); the proportion of the mechanical energy that can be saved through pendulum-like mechanisms [33, 34]:

$$ R=\frac{W_v+{W}_F-{W}_{EXT}}{W_v+{W}_F}\times 100 $$

where *W*_v_ and *W*_f_ are the vertical and horizontal components of the *E*_CM_, respectively.

The efficiency of positive work production (*η*_*loco*_) was calculated from the ratio of the total positive mechanical cost of locomotion (*W*_*TOT*_, J kg^− 1^ m^− 1^) to the metabolic cost of locomotion (*C*_*met*_):

$$ {\eta}_{loco}=\frac{W_{TOT}}{C_{met}} $$

### Statistical analyses

Data were analysed using a linear mixed-effect model, with speed of level walking (0.8, 1.0, 1.2, 1.4, and 1.6 of customary speed) or gradient of declined walking (0°, 5°, 10°) and foot attachment (*hy*A-F, *rig*A-F) as factors. Statistical analyses were conducted using the *nlme* [35] and *car* [36] packages in R [37]. A random intercepts model with subjects specified as a random factor was used to control for their associated intra-class correlation [38] and the repeated measures design of the experiment. Model fit was assessed using Chi-square tests on the log-likelihood values to compare different models. In instances where unequal variance was apparent for one of the predictor variables, the variable was log-transformed or, if this did not improve the variance structure, the model was updated by generating a constant variable structure for the predictor variable in question [39] using the *varIdent* function in the *nlme* package [35]. Level of significance for all statistical analyses was accepted at *p* < 0.05. *P*-values between 0.05-0.10 were considered to indicate a statistical trend.

## Results

The key focus of the study was to determine how use of a *hy*A-F compared to *rig*A-F affected various gait outcome measures. As such, although the significant walking speed and surface gradient main effects are indicated within the text, the resulting effects are not described. Similarly, because there were no significant interactions found between foot attachment type and walking speed or surface gradient for any of the outcome variables analysed (*p* > 0.05), there is no further mention of interaction effects.

The group mean freely chosen comfortable (customary) walking speed was 0.98 (± 0.04, s.e.m., range 0.68 to 1.08) m s^− 1^.

### Metabolic cost of locomotion

**Fig. 1:**
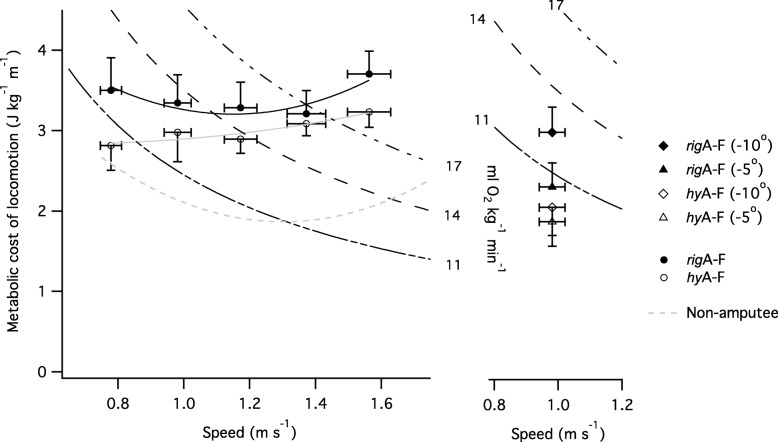
Metabolic cost of locomotion across the different speeds and different angles of decline for each foot attachment conditions. Iso-metabolic power lines (representing effort) at 11, 14 and 17 ml O_2_ kg^− 1^ min^− 1^ are presented. Note that this Fig. highlights absolute speed rather than percentage of customary; the value plotted is the mean absolute speed for each category of relative customary speed category (0.8, 1, 1.2, 1.4 and 1.6 of customary speed, NB group mean customary speed was 0.98 m s^− 1^). The metabolic cost of walking (dashed line) in non-amputees, previously reported in the literature are plotted for comparison [40]. Data are mean ± SEM

*C*_met_ across the different speeds and different angles of decline (at customary speed) for each foot attachment condition are shown in Fig. 1. When walking on the level, *C*_met_ was significantly affected by attachment condition (χ^2^(1) = 11.62, *p* < 0.001) but not by walking speed (χ^2^(4) = 7.66, *p* = 0.11). *C*_met_ was lower (by 11.8 ± 2.5%, averaged across all level speeds, ± s.e.m.) during level walking using the *hy*A-F compared to the *rig*A-F prosthesis. For both attachment types, *C*_met_ was higher than previously published data on non-amputees (Fig. 1; [40]). For gradient walking, both the gradient (χ^2^(2) = 23.22, *p* < 0.001) and attachment condition (χ^2^(1) = 15.61, *p* < 0.001) had a significant effect on *C*_met_. *C*_met_ was lower (by 20.2 ± 3.4%, averaged across three gradients at the customary speed, ± s.e.m.) during gradient walking when using the *hy*A-F compared to the *rig*A-F.

Figure 1 shows the iso-metabolic power lines (representing metabolic power, which is related to the perceived effort) at 11, 14 and 17 ml O_2_ kg^− 1^ min^− 1^. It shows that for a metabolic power of 14 ml kg^− 1^ min^− 1^ an amputee can walk at 1.18 m s^− 1^ with the *hy*A-F but only at 1.09 m s^− 1^ with the *rig*A-F (for the same level of effort). Similarly, for a metabolic power of 17 ml kg^− 1^ min^− 1^ an amputee can walk at 1.45 m s^− 1^ with the *hy*A-F but only at 1.37 m s^− 1^ with the *rig*A-F (for the same level of effort). These estimates are based on the intersections of the metabolic cost of locomotion versus speed curves and the iso-metabolic power curves. For comparison, a non-amputee can walk at 1.63 m s^− 1^ and 1.80 m s^− 1^ for a level of effort of 14 ml kg^− 1^ min^− 1^ and 17 ml kg^− 1^ min^− 1^, respectively.

### 3D trajectories of the BCoM and inter-limb symmetry

**Fig. 2:**
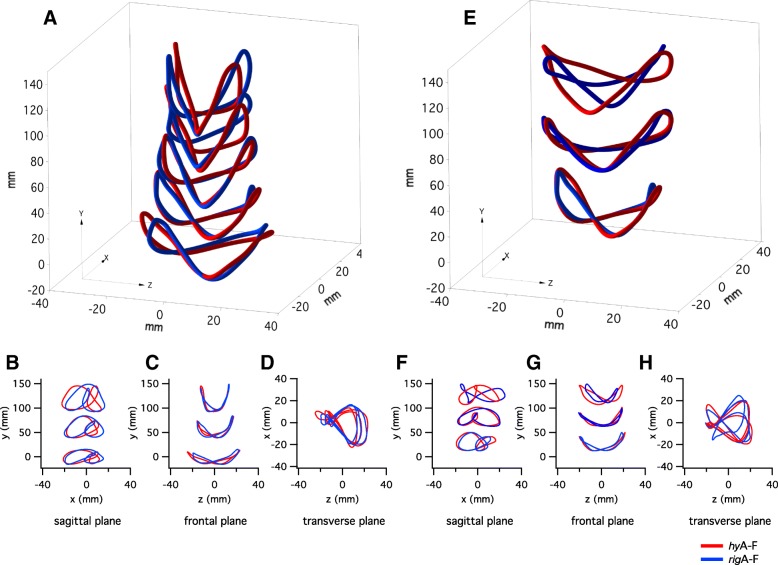
Average trajectories of the BCoM during (**a**-**d**) level walking and (**e**-**h**) walking at 100% of the customary speed during level or decline walking. The average trajectories represent right-legged trans-tibial amputees [where participants were left-leg amputees, the BCoM trajectories were reflected in the sagittal (x-y) plane]. **a** 3D-BCoM trajectories during level walking at 80–160% customary speed, and **b**-**d** 2D-BCoM trajectories during level walking at 80, 120 and 160% customary speed, for each prosthetic foot attachment condition (red *hy*A-F, blue *rig*F; see also legend). **e** 3D-BCoM trajectories, and **f**-**h** 2D-BCoM trajectories, walking at 100% of the customary speed on level, 5° decline and 10° decline. On the plots (**a**, **b**, **c**, **e**, **f**, **g**), for each condition (speed or decline), trajectories are incrementally offset on the craniocaudal axis (y-axis) by 30 mm for level walking and 50 mm for decline walking, for clarity. On the 3D-plots, the subject is walking from lower-left to upper-right along the anteroposterior axis (x-axis); hence positive medio-lateral (‘z’) displacement represents rightwards movement and negative ‘z- displacement represents leftwards movement. Movies of the rotation of the 3D trajectories of the BCoM are available as Additional files (see 10.5518/272)

Figure 2 shows the average BCoM profiles during level walking at different speeds (Fig. 2 a-d) and level/decline walking at customary speed (Fig. 2 e-h) whilst using each foot attachment type (movies of the rotation of the 3D trajectories of the BCoM are available in the following data repository: 10.5518/272). For all conditions the BCoM trajectories exhibited notable inter-limb asymmetry in all planes, though asymmetry was greatest in the horizontal plane (x-z plane). Note, in the horizontal plane (x-z plane) (Fig. 2 d), there is a large loop corresponding to the prosthetic-limb stance phase (rightwards direction) and a smaller loop corresponding to the intact-limb stance phase (leftwards direction). This is consistent with the low *SI*_x_ and *SI*_z_ inter-limb symmetry (see below). The mediolateral (z-axis) displacement of the BCoM decreased with increasing speed and the craniocaudal (y-axis) displacements increased with increasing speed; with anteroposterior (x-axis) displacement being more or less constant across speeds (Fig. 2 a). Displacements in each plane were similar for the *hy*A-F and *rig*A-F devices.

**Fig. 3:**
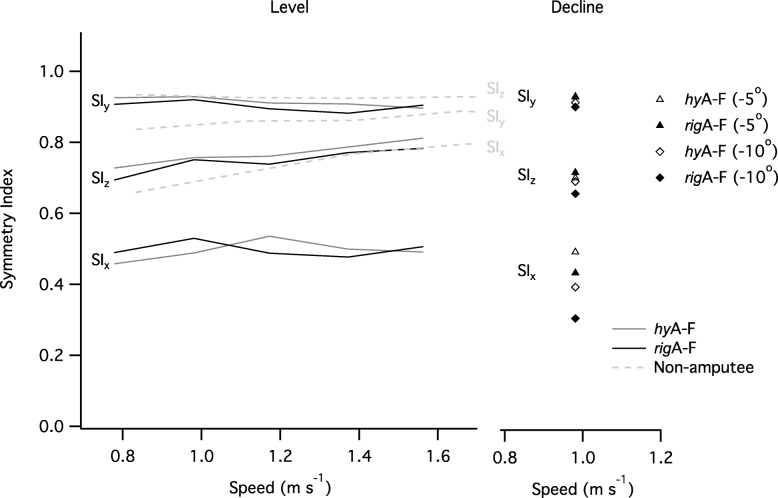
Symmetry indices (SI) as functions of walking speed and gradient for two types of prosthetic. The symmetry indices (dashed line) in non-amputees, previously reported in the literature are plotted for comparison (Minetti et al., 2011 [30]). Error bars have been omitted for clarity. Note that this Fig. highlights absolute speed rather than percentage of customary; the value plotted is the mean absolute speed for each category of relative customary speed category (0.8, 1, 1.2, 1.4 and 1.6 of customary speed: NB group mean customary speed was 0.98 m s^− 1^)

For level walking, the symmetry between intact and prosthetic -limb steps (*SI*) was highest in the vertical (craniocaudal) axis, but neither walking speed (χ^2^(2) = 8.50, *p* = 0.07), nor attachment type (χ^2^(1) = 3.29, *p* = 0.07) had a significant effect on *SI*_y_. Symmetry was lowest in the progression (anteroposterior) axis (*SI*_x_) but neither walking speed (χ^2^(2) = 2.24, *p* = 0.69), nor attachment type (χ^2^(1) = 0.07, *p* = 0.79) had a significant effect on *SI*_x_. However, symmetry in the medio-lateral axis (*SI*_*z*_), was significantly affected by the speed of walking (χ^2^(4) = 19.48, *p* < 0.001) and attachment type (χ^2^(1) = 3.92, *p* < 0.05). *SI*_*z*_ was higher (better) when using the *hy*A-F compared to the *rig*A-F, though the differences were not significant (SI_z_; β = − 0.02, t_42_ = − 1.91, *P* = 0.06; Fig. 3).

For gradient walking, the BCoM underwent greater forward excursion in the anteroposterior axis (x-axis), compared to level walking, and the effect was higher at steeper gradients. During walking on a 5° decline the BCoM trajectories were similar for the *hy*A-F and *rig*A-F devices. However, walking on a 10° decline resulted in a striking difference in the BCoM trajectory between the two types of attachment. This difference indicates that inter-limb symmetry in the anteroposterior axis (x-axis) was lower using the *rig*A-F compared to using the *hy*A-F (Fig. 3). Gradient had a significant effect on the symmetry in all three axes (*SI*_x_; χ^2^(2) = 13.19, *p* = 0.001; *SI*_y_; χ^2^(2) = 16.23, *p* < 0.001; *SI*_z_ (χ^2^(2) = 7.94, *p* = 0.001), but attachment type had no effect (*SI*_x_, *p* = 0.31; *SI*_y_, *p* = 0.96; *SI*_z_, *p* = 0.16). Although the differences were not statistically significant, *SI*_*z*_ was consistently higher (better) when using the *hy*A-F compared to *rig*A-F (trend only; Fig. 3).

### External, internal and total mechanical work

**Fig. 4:**
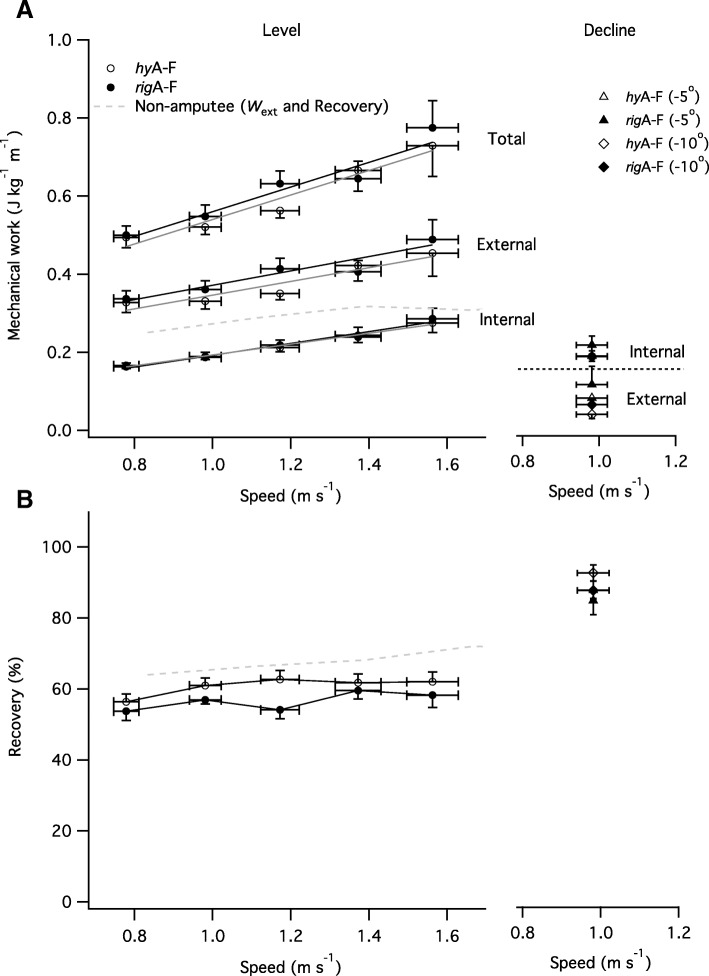
**a** External, internal and total mechanical energy during level and decline walking for *rig*A-F and *hy*A-F prostheses. Regression lines for the relationships between mechanical work and speed are shown in grey (*rig*A-F) and black (*hyA-F*). **b** The interchange of potential and kinetic energies of the BCoM, quantified as the recovery index (%), are shown for level and decline walking for *rig*A-F and *hy*A-F prostheses. Data are mean ± SEM. The external mechanical work and recovery index (dashed lines) in non-amputees, previously reported in the literature are plotted for comparison (Minetti et al., 2011). Note that this figure highlights absolute speed rather than percentage of customary; the value plotted is the mean absolute speed for each category of relative customary speed category (0.8, 1, 1.2, 1.4 and 1.6 of customary speed: NB group mean customary speed was 0.98 m s^− 1^)

For level walking, *W*_EXT_ increased with increasing speed (χ^2^(4) = 36.90, *p* = 0.001) and was generally higher with the *rig*A-F compared with *hy*A-F, however this difference was neither significant nor consistent across all speeds (χ^2^(1) = 3.45, *p* = 0.06; trend only; Fig. 4a). *W*_INT_ increased with walking speed (χ^2^(4) = 91.57, *p* < 0.001), but attachment type had no effect (χ^2^(1) = 0.08, *p* = 0.77). *W*_TOT_ increased with increasing speed (χ^2^(4) = 39.56, *p* < 0.001) and was generally lower when using the *hy*A-F compared with the *rig*A-F, however, the difference was not significant (χ^2^(1) = 2.70, *p* = 0.10; trend only; Fig. 4a). The type of prosthetic had a significant effect on the partitioning of *W*_INT_ between the lower left (non-amputated; **χ**^**2**^ (1) = 13.55, *p* = 0.0002) and right (amputated; **χ**^**2**^ (1) = 35.01, *p* < 0.0001) limbs. The non-amputated lower limb represented a higher proportion of *W*_INT_ than the amputated limb and the difference between the two limbs was greatest when walking with *rig*A-F compared with *hy*A-F (β = 0.22, t_42_ = 6.83, *P* < 0.0001).

For gradient walking, *W*_INT_ was unaffected by gradient (χ^2^(2) = 2.32, *p* = 0.31); and attachment type (χ^2^(1) = 0.98, *p* = 0.32) but W_EXT_ and *W*_TOT_ were significantly affected by both gradient (*W*_EXT_ χ^2^(2) = 66.86, *p* < 0.001; *W*_TOT_ χ^2^(2) = 88.23, *p* < 0.001) and attachment type (*W*_EXT,_ χ^2^(1) = 4.87, *p* = 0.03; *W*_TOT_, χ^2^(1) = 6.58, *p* = 0.01). W_EXT_ and *W*_TOT_ were higher when using the *rigA-F* compared to the *hy*A-F. The type of prosthetic had a significant effect on the partitioning of *W*_INT_ between the lower left (non-amputated; **χ**^**2**^ (1) = 4.50, *p* = 0.03) and right (amputated; **χ**^**2**^ (1) = 15.44, *p* = 0.0001) limbs. The non-amputated lower limb represented a higher proportion of *W*_INT_ than the amputated limb and the difference between the two limbs was greatest when walking with *rig*A-F compared with *hy*A-F (β = 0.35, t_42_ = 2.74, *P* = 0.011).

For level walking, the recovery index (*R*) was dependent on speed of walking (χ^2^(4) = 10.20, *p* = 0.04) and attachment type (χ^2^(1) = 12.11, *p* < 0.001, Fig. 4b). Walking using the *hy*A-F resulted in a higher *R* (by approximately 4%) compared with the *rig*A-F (β = − 4.19, t_42_ = − 3.49, *P* = 0.001). For gradient walking *R* was affected by gradient (χ^2^(2) = 64.49, *p* < 0.001) and attachment type (χ^2^(1) = 5.60, *p* = 0.02, Fig. 4b). *R* was higher when using the *hy*A-F (by approximately 4%) compared with *rig*A-F device.

### Locomotor efficiency (*η*_*loco*_)

**Fig. 5:**
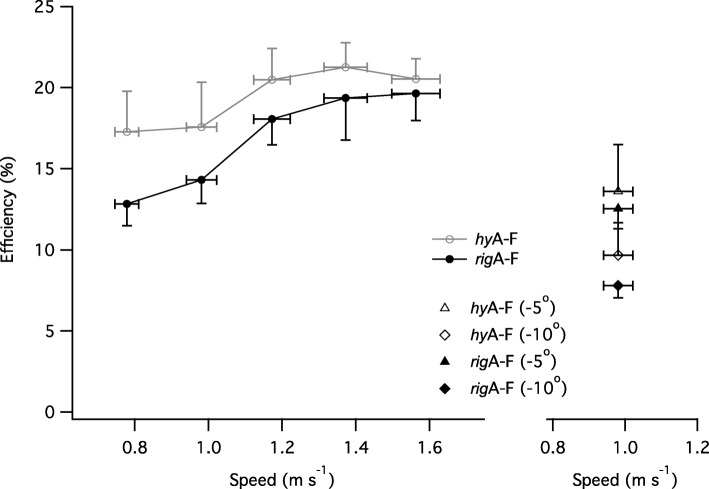
The efficiency of positive work production during level and decline walking using either *rig*A-F and *hy*A-F prosthesis. Data are mean ± SEM. Note that this figure highlights absolute speed rather than percentage of customary; the value plotted is the mean absolute speed for each category of relative customary speed category (0.8, 1, 1.2, 1.4 and 1.6 of customary speed: NB group mean customary speed was 0.98 m s^− 1^)

For level walking, *η*_*loco*_ was dependant on walking speed (χ^2^(4) = 15.54, *p* = 0.004) and attachment type (χ^2^(1) = 10.39, *p* = 0.001). *η*_*loco*_ was higher when using the *hy*A-F compared with *rig*A-F. For gradient walking, gradient (χ^2^(2) = 10.19, *p* = 0.006) and attachment type (χ^2^(1) = 9.62, *p* = 0.002) had a significant effect on *η*_*loco*_ (Fig. 5). Walking on a 10° decline, using the *rig*A-F resulted in a significantly lower *η*_*loco*_ (β = − 1.85, t_21_ = − 6.44, *P* < 0.001) compared to using the *hy*A-F.

## Discussion

Results indicate that use of a DRF that has an ankle mechanism providing a small range of hydraulically controlled passive articulation at the point of attachment should significantly reduce the energy expenditure per metre travelled in comparison to using the same DRF that has no ankle mechanism. Accordingly, for a given rate of metabolic energy expenditure it should be possible to walk at a faster speed when the DRF being used incorporates a small hydraulic ankle mechanism. For example, using the *hy*A-F it is possible to walk at speeds that are approximately 6–8% higher compared to those using a *rig*A-F device, for the same level of effort. An increase in UTAs’ freely chosen walking speed, following switching from using a *rig*A-F to a *hy*A-F, has been a consistent finding in previous studies [1, 20, 41, 42].

The analyses of segmental and whole-body mechanical energetics indicate that the reduction in metabolic energy expenditure per metre travelled when using the *hy*A-F was due to a higher recovery index (interchange between BCoM potential and kinetic energy), resulting in a higher locomotor efficiency (i.e. greater ratio of positive mechanical work to metabolic cost of locomotion), in comparison to that using the *rig*A-F. Importantly, the reduced metabolic cost of locomotion and increased efficiency when using the *hy*A-F occurred despite the fact that a *hyA-F* has a mass approximately 400 g higher than a *rig*A-F (which is expected to increase the cost of swinging the leg; [43]) and that the hydraulic dashpot incorporated within the ankle device will dissipate energy.

There was no difference between foot attachment conditions in the amount of internal mechanical work done (i.e. positive increments in the kinetic energy of each segment) but, during level walking, the external mechanical work (sum of the instantaneous kinetic and potential energy of the BCoM) was generally lower when using the *hy*A-F compared to *rig*A-F although differences were not significant (*p* = 0.06; i.e. trend only). This trend would suggest that there were reduced and/or fewer incremental changes in BCoM kinetic and potential energy when using the *hy*A-F compared to *rig*A-F, which could be reflective of smoother (less jerky) and/or smaller changes in BCoM trajectory. Inter-limb symmetry, was found to be consistently higher during level walking in the medio-lateral (*p* = 0.05) and craniocaudal (vertical) (*p* = 0.07, trend only) axes when using the *hy*A-F compared to *rig*A-F. With both types of attachment, inter-limb symmetry in all axes was significantly reduced as the gradient of descent increased; however, symmetry in the medio-lateral was found to be consistently higher (non-significant) when using the *hy*A-F compared to the *rig*A-F. These inter-limb symmetry improvements when using the *hy*A-F indicate BCoM excursion during prosthetic and intact limb stance phases/steps became more symmetrical, which suggests that use of the *hy*A-F also reduced the compensatory actions needed to walk. The differences in gait symmetry were most striking when walking on a 10° slope. Previous research has shown that when amputees descend slopes using a prosthesis incorporating a foot that is rigidly attached to the shank pylon (i.e. has no ‘ankle’), compensatory knee flexion increases [18, 44]. The increased knee flexion causes increased residuum loading, which in turn leads to poorer knee stability [18]. Hence the inclusion of a small ankle unit into such prostheses has been shown to lead to improvements in slope descent [45]. It is thus likely that the improvement in inter-limb symmetry on the 10° slope when walking with *hy*A-F compared to *rig*A-F, is reflective of a reduction in the magnitude or number of gait compensations used. This helps explain, at least in part, why the metabolic energy expenditure per metre travelled was found to be reduced using the *hy*A-F compared to *rig*A-F. The latest *hy*A-F devices now incorporate a microprocessor that automatically alters the hydraulic resistance in accordance with the speed of walking and the surface gradient. Future work is required to determine if and how the mechanical and metabolic energies of locomotion are affected by using such feet.

In previous work, we have shown that compared to using a DRF with rigid attachment (no ‘ankle’), use of a *hy*A-F leads to bodyweight being transferred onto the prosthetic limb in a smoother less faltering manner with accompanying smaller reduction in BCoM forward velocity [20, 41], and to a reduction in compensatory intact-limb stance phase joint work per metre travelled [1]. It was deduced that these findings resulted from the device reducing the ‘braking effect’ exerted by the prosthetic limb during early stance rather than the device enhancing the foot’s ability to return (recoil) spring energy [41]. Such ‘braking effect’ is because the foot’s heel region and/or keel(s) deform/deflect during limb loading, which delays or slows the shank’s forward rotation over the foot and either extends the period in which the ground reaction force is directed posteriorly or increases the magnitude of this posteriorly directed force [41, 46–48]: either of which will decelerate the BCoM forwards velocity. The findings in the present study are in agreement which such an interpretation, i.e. the benefit of using a *hy*A-F is that it diminishes the magnitude and/or speed of BCoM trajectory changes; as evidenced by the finding of higher recovery (and trend of less external and total mechanical work being done) and improved inter-limb symmetry in BCoM excursion when using the *hy*A-F compared to *rig*A-F. The higher recovery index (and reduction in external and total mechanical work) and higher inter-limb symmetry led to the net metabolic cost of locomotion being reduced and to gait efficiency being increased. In our previous paper, we showed that when using a *hy*A-F compared to *rig*A-F, there is a smoother and more rapid progression of the ground reaction force under the prosthetic foot, which meant the prosthetic limb exerting a reduced ‘braking effect’, which in turn reduced the compensatory intact-limb stance phase work that would have otherwise been required to ‘push’ the BCoM onto the prosthetic limb [1]. This reduced ‘braking effect’ and associated reduction in ‘push’ [1] highlights the BCoM went through reduced acceleration changes, which is concomitant with the present study’s findings of less external mechanical work being done on the BCoM and improved inter-limb symmetry in BCoM excursion for prosthetic and intact limb steps, when using the *hy*A-F compared to *rig*A-F. For both level and gradient walking, the intact lower limb represented a higher proportion of *W*_INT_ than the amputated limb and the difference between the two limbs was greatest when walking with *rig*A-F compared with *hy*A-F lower left. The relatively higher intact lower limb *W*_INT_ when using the *rig*A-F compared to *hy*A-F was likely a consequence of the intact limb’s ankle musculature doing more work to ‘push’ the BCoM onto the prosthetic limb because of the increased ‘braking effect’ from the prosthetic limb when using the *rig*A-F compared to *hy*A-F.

### Biomechanics and energetics of unilateral trans-tibial amputee (UTA) participants compared with able-bodied subjects (reported in the literature)

Across the range of speeds studied, the metabolic cost of locomotion in our UTA participants was higher compared to that reported for able-bodied subjects (e.g. [40], see Fig. 1), and comparable to previous data on UTAs using devices similar to those used by participants in the present study (e.g. [49]). As with previous studies, the speed at which the net metabolic cost of locomotion was minimal (also known as the ‘optimum speed of walking’) was lower than that reported for able-bodied subjects [49, 50]. Unsurprisingly, participants’ gait exhibited a high degree of asymmetry, particularly in the horizontal (x-z) plane. Even healthy, able-bodied subjects exhibit a degree of gait asymmetry [30], but the extent is much lower than that found in the present study [see Fig. 3 where data from able bodied subjects [30] are plotted for comparison]. The relatively high gait asymmetry found in the present study is likely related to lower-limb anatomical asymmetries, and may contribute to the higher net metabolic cost of locomotion in amputees. Participants’ recovery index was similar to that reported for able-bodied subjects (Fig. 4b; [33]) and higher (especially at low walking speeds) than has previously been reported for UTAs [49]. There was no relationship between recovery index and speed in the present study, which corroborates previous work in young and older able-bodied adults [33]. In contrast, an earlier study found that recovery index increased with speed [49]; however, the speeds and the range of speeds used in this earlier study (0.4 to 0.9 m s^− 1^) are lower than those used in the present study. Across the range of speeds investigated, efficiency of locomotion ranged from 12 to 21%, which is much lower than that previously found (18 to 32%) in able-bodied adults during level walking [33]. However, the total mechanical work by participants is similar to that reported for able bodied subjects [33] indicating that the lower efficiency of locomotion was mainly due to the higher metabolic cost of locomotion.

Potential limitations of the present study include the limited time participants had to familiarise themselves to walking with the *hy*A-F, which was between 30 and 40 min. However, given that the order in which the two foot attachment types (*rig*A-F, *hy*A-F) was worn was counterbalanced across participants, we do not believe the limited familiarisation time affected the conclusions made. Another potential limitation was that those with a higher preferred walking speed would have had a higher range of absolute speeds (0.8 to 1.6 of preferred walking speed) than someone with a lower preferred walking speed. For example, the participant who had the highest preferred speed of 1.08 m s^− 1^ had a speed range of 0.864 to 1.728 m s^− 1^, while the participant who had the lowest preferred speed of 0.68 m s^− 1^ had a speed range of 0.544 to 1.088 m s^− 1^. Although these speed ranges represent the same percentage of an individual’s preferred speed, the differences in the absolute speed ranges across participants, may partly explain the relatively large inter-subject variability in some of the outcome variables. Finally, the relatively small sample size of 9 UTA is a potential limitation because, although this sample size is not untypical for studies involving lower-limb amputees, it did limit the study’s statistical power; thus findings should be interpreted accordingly.

## Conclusions

Findings indicate that compared to using a DRF that allows no articulation at the point of attachment (*rig*A-F), use of the same DRF that incorporates an ‘ankle’ mechanism providing a small range of hydraulically controlled passive articulation at the point of attachment (*hy*A-F), significantly improved the interchange between the mechanical energies of the BCoM (recovery index) and lead to a significant reduction in metabolic energy cost per metre travelled, and hence an increase in gait efficiency. It also improved inter-limb symmetry highlighting the improvements in gait were related to a lessening of the kinematic compensations evident when using the *rig*A-F. These findings suggest that use of an Echelon prosthetic foot (*hy*A-F) will provide clinically meaningful benefits to UTAs’ everyday locomotion.
